# The regulation of vacuole morphology in stigma papilla cells is involved in water transfer to pollen in *Arabidopsis thaliana*

**DOI:** 10.1007/s00497-025-00525-1

**Published:** 2025-06-06

**Authors:** Kazuki Fukushima, Maki Hayashi, Masao Watanabe

**Affiliations:** Graduate School of Life Sciences, Tohoku University, Sendai, Miyagi 980-8577 Japan

**Keywords:** *Arabidopsis thaliana*, Live-cell imaging, Papilla cells, Pollen hydration, Vacuole

## Abstract

**Key message:**

The stigma papilla cells of *Arabidopsis thaliana* control water transport to pollen by regulating the morphology of vacuoles in papilla cells after pollination.

**Abstract:**

Pollen hydration is the first crucial response after pollination for successful fertilization. In the Brassicaceae family, papilla cells on the stigma supply water to pollen. In pollinated papilla cells, cellular responses essential for pollen hydration are induced. However, it remains unclear how papilla cells release water from inside the cells to the pollen. Here, we set up a live-cell imaging system for observing vacuole dynamics in *Arabidopsis thaliana* papilla cells and investigated the role of vacuole morphology in these cells in the regulation of water transfer to pollen. Before pollination, vacuoles in the papilla cells changed their morphology through fusion and constriction; however, after pollination, they formed larger vacuoles and exhibited reduced movement. Additionally, when the morphological variation of vacuoles in the papilla cells was inhibited by wortmannin treatment, the pollen hydration rate decreased in a concentration-dependent manner. In contrast, the vacuoles tended to be less constricted even before pollination and showed less variation than wild-type after pollination in Rho-like GTPase from plants 2 (ROP2) mutant papilla cells, where the pollen hydration rate is faster. We propose that the regulation of vacuole morphology in papilla cells is involved in water transfer to pollen during pollination.

**Supplementary Information:**

The online version contains supplementary material available at 10.1007/s00497-025-00525-1.

## Introduction

Pollen hydration is the first crucial response induced by male–female interactions after pollination. The flowers of Brassicaceae plants have a dry stigma and long, elongated cells called papilla cells on the tip of the stigma (Dickinson 1995). When pollen attaches to papilla cells in pollination, a specialized “foot” structure is formed to anchor pollen to papilla cells (Elleman and Dickinson 1990). Because pollen hydration does not occur without the foot structure, it is believed that pollen receives water and essential factors required for pollen hydration, and subsequent germination of the pollen tube, from papilla cells through the structure (Chapman and Goring 2010). Following hydration, the pollen tube germinates from the pollen grain and elongates through the stigma toward the ovules. Therefore, because pollen hydration is required for pollen tube germination and elongation, it is a critical response that determines the ultimate success of fertilization.

During pollination in *Brassica rapa*, a Brassicaceae species, the repeated expansion and contraction of pollen as it hydrates on papilla cells, or bursting of pollen after it has expanded, suggests that for normal pollen tube germination, the papilla cells must correctly control the transfer of water to the pollen (Hiroi et al. 2013). Studies have revealed that cellular responses, such as actin remodeling (Iwano et al. 2007; Rozier et al. 2020), vesicle transport (Elleman and Dickinson 1990, 1996; Safavian and Goring 2013; Macgregor et al. 2024), increased calcium ion concentration in the cytoplasm of papilla cells (Iwano et al. 2014), and decreased reactive oxygen species (ROS) concentration in the cytoplasm of papilla cells (Liu et al. 2021; Zhang et al. 2021), occur in papilla cells after compatible pollination of *Brassica* and *Arabidopsis* plants. These responses are required for pollen hydration (Abhinandan et al. 2022). However, the mechanism by which papilla cells release water from the inside of the cells to pollen remains unclear.

Plants increase cell volume through water uptake during development. In this process, it is believed that plant cells maintain a constant solute concentration in the cytoplasm by taking up most of the volume-enhancing water into the vacuole (Taiz 1992). In Brassicaceae plants, pollen hydration is completed in about 15–90 min after pollination (Dickinson 1995; Hiroi et al. 2013; Wang et al. 2017; Rozier et al. 2020). This dramatic pollen hydration requires a source of sufficient water. Therefore, vacuoles, which contain abundant water in the papilla cells, are hypothesized to be the source of water for the pollen. Recently, plasma membrane aquaporins (PIPs), which promote water movement across the plasma membrane, have been shown to contribute to pollen hydration (Windari et al. 2021; Liu et al. 2025). Because the water transport ability of aquaporins is passive, the osmotic pressure difference between papilla cells and pollen is required as a driving force to move water from the papilla cells to the pollen. However, the mechanism regulating osmotic pressure in papilla cells remains unclear.

A previous study observing papilla cells of *B. rapa* showed that there were different sizes of vacuoles in the apical region of papilla cells (Iwano et al. 2007). That is, there were large, small, and elongated (tubular) vacuoles in unpollinated papilla cells. The large vacuoles were located at the center of the papilla cells, surrounded by the small and tubular vacuoles. Furthermore, connections between the tubular and large vacuoles were observed, suggesting that fission of the large vacuoles could produce the tubular vacuoles in papilla cells. After compatible pollination, the large central vacuoles tended to elongate towards the site of pollen attachment, while the tubular vacuoles also elongated alongside the large vacuoles. These observations suggest that the dynamics of the large central vacuoles in papilla cells might be important for water transport from papilla cells to pollen (Iwano et al. 2007). However, the details of how vacuole dynamics contribute to water transfer remain unclear.

In this study, we set up a live-cell time-lapse imaging system with confocal microscopy to analyze vacuole dynamics and dissect their role in water transfer from papilla cells to pollen. To visualize the vacuoles, we generated transgenic plants of *Arabidopsis thaliana* (also a Brassicaceae species) that express green fluorescent protein (GFP) targeted to the tonoplast. We characterized the pattern of the vacuole dynamics in the transgenic plants in unpollinated and pollinated papilla cells using image analysis methods.

## Materials and methods

### Plant materials and growth conditions

The *Arabidopsis thaliana* plants used in this study were Columbia-0 (Col-0) ecotypes. The T-DNA mutant *rop2-1* (SALK_055328C) was obtained from the Arabidopsis Stock Centre (ABRC). All plants were grown in soil under long-day conditions (16 h light/8 h dark) at 23 °C and 60% humidity for 3–5 weeks.

### Plasmid construction

The tonoplast-targeting sequence (TTS) used to create the *TTS::GFP* construct was derived from CBL2 (Tang et al. 2012). *TTS::GFP* was amplified using specific primers (5′-ATGTCGCAGTGCGTTGACGG-3′, 5′-TTACTTGTACAGCTCGTCCATGCC-3′) and introduced into the pRI101-AN vector (TaKaRa) using In-fusion cloning (Clontech) to create the *35Spro-TTS::GFP*/pRI101-AN construct.

For the construction of *AtS1pro-TTS::GFP*, first, the *AtS1* stigma-specific promoter (Dwyer et al. 1992, 1994) was amplified using specific primers (5′-CATGATTACGAATTCTCGAAATACATCGAGAAG-3′, 5′-ACCGAGCTCGAATTCCTTCACGACTTTCTTTCTTATGC-3′) and inserted into the pCAMBIA1300 vector (CAMBIA) by In-Fusion to generate *AtS1pro*/pCAMBIA1300. *TTS::GFP* was then cloned from *35Spro-TTS::GFP*/pRI101-AN using primers 5´-AAGAAAGTCGTGAAGATGTCGCAGTGCGTTGACGG-3´ and 5´-GGCCAGTGCCAAGCTGATCTAGTAACATAGATGACACCGCG-3´. This fragment was inserted under the *AtS1* promoter in the pCAMBIA1300 vector by In-Fusion to generate *AtS1pro-TTS::GFP*/pCAMBIA1300.

### Plant transformation

The *35Spro-TTS::GFP*/pRI101-AN, *AtS1pro-TTS::GFP*/pCAMBIA1300, *AtS1pro-lifeact::tdTomato*/pRI909, and *UBQ10pro-PIP2a::mCherry*/pCMU (plasma membrane-localized protein PIP2a fused to red fluorescent protein mCherry expressed under *UBQ10* ubiquitous promoter) constructs were transformed into *Agrobacterium tumefaciens* GV3101 strain. *Agrobacterium* was infected into Col-0 or *rop2-1* using the floral-dip method (Clough and Bent 1998). Plants expressing *35Spro-TTS::GFP*/pRI101-AN, *AtS1pro-lifeact::tdTomato*/pRI909, or *UBQ10pro-PIP2a::mCherry*/pCMU were generated in the Col-0 background. Plants expressing both of *35Spro-TTS::GFP*/pRI101-AN and *AtS1pro-lifeact::tdTomato*/pRI909 were generated by crossing. Plants expressing *AtS1pro-TTS::GFP*/pCAMBIA1300 were generated in the *rop2-1* mutant background. T_1_ seedlings were selected on half-strength MS growth plates containing carbenicillin and kanamycin for *35Spro-TTS::GFP*/pRI101-AN, *AtS1pro-lifeact::tdTomato*/pRI909, and *UBQ10pro-PIP2a::mCherry*/pCMU or hygromycin for *AtS1pro-TTS::GFP*/pCAMBIA1300. T_1_, T_3_, and T_4_ plants were used in the experiments.

### Chemical treatment of stigma papilla cells

E-64d ((2s, 3s)-*trans*-epoxy-succinyl-L-leucylamido-3-methylbutane ethyl ester, Peptide Institute), PDMP (D-L-*threo*−1-phenyl-3-decanoyl amino-3-morpholino-1-propanol, Enzo Life Sciences), or wortmannin (Fujifilm Wako Pure Chemical Corporation) dissolved in DMSO with 0.025% Tween 20. Stigma papilla cells were treated with 0.5 µl of the solutions dispensed with a micropipette. The chemical-treated stigmas were air dried on 1% agar for at least 30 min. After this chemical treatment, live-cell time-lapse imaging of vacuoles in the papilla cells and pollen hydration assay were performed. Col-0 pistils were used in all experiments.

### Live-cell time-lapse imaging of stigma papilla cells

Pistils were obtained from flower buds that were emasculated and covered with plastic wrap before the day of the experiment. Pistils with fully elongated papilla cells were inserted into 1% agar and mounted on glass-bottom dishes with the papilla cells placed in contact with the cover glass. Time-lapse imaging of vacuoles in papilla cells was performed using laser-scanning confocal microscopy (LSM710, Zeiss) with a 40 × oil objective (Zeiss). Three or five z-stack images (4 µm thickness) were obtained from the surface of the tonoplast signal to check that the samples had not been moved. Images of tonoplast in unpollinated papilla cells were obtained as single-channel (GFP excited at 488 nm) image stacks at 10-s time intervals and five z-stacks with a 1-µm z-step size. Images of tonoplast and actin in unpollinated papilla cells were obtained as double-channel (GFP excited at 488 nm and tdTomato excited at 561 nm) image stacks 3 min after pollination at 10-s time intervals and three z-stacks with a 2-µm z-step size. Images of tonoplast in pollinated papilla cells with pollen were obtained as double-channel (GFP excited at 488 nm and mCherry excited at 561 nm) image stacks 3 min after pollination at 10-s time intervals and three z-stacks with a 2-µm z-step size. *UBQ10pro-PIP2a::mCherry*-expressing pollen of Col-0 background was applied to the papilla cells by hand pollination. The medial z-slice image (second of three or third of five) was used for all representative fluorescence images shown in the figure parts and analyzed for vacuole dynamics.

To count the number of vacuole constrictions, 10-min movies (60 frames at 10-s time intervals) of tonoplast signals were observed. The number of vacuole constrictions was counted as the number of appearances of vacuole borders completely across papilla cells (Supplementary Fig. S1a).

### Image analysis of vacuole movement

To quantify changes of the vacuoles in papilla cells, correlation analysis of images was performed with reference to Higaki et al. (2006). Briefly, the normalized correlation coefficient between the consequence frames is defined as:

1 $$\begin{array}{c}\frac{\sum_{x, y}\left(A\left(x, y\right)-\overline{A }\right)\left(B\left(x, y\right)-\overline{B }\right)}{\sqrt{{\sum_{x,y}(A\left(x, y\right)-\overline{A })}^{2}{\sum_{x,y}(B\left(x, y\right)-\overline{B })}^{2}}}\end{array}$$

where $A(x, y)$ and $B(x, y)$ are respective pixel values of the images, and $\overline{A }$ and $\overline{B }$ are mean values of pixel values of the images. If the two images are similar, the normalized correlation coefficient value is close to 1. The 3 × 3 median filter processed all images to remove noise using Fiji (Schindelin et al. 2012). After the noise was removed, the histogram of the images was adjusted so that the 97th percentile of intensity was the maximum. The values were calculated from two images, one at the initiation of live imaging and one at each time point, to provide an indication of the extent to which the image had changed from the image at the start of imaging.

### Pollen hydration assay

Pistils with fully elongated papilla cells, which were obtained from emasculated flower buds, and anthers were mounted on coverslips with double-sided tape and fixed with surgical tape (3M). The cut edge of the stigmas was covered with a piece of 1% agar to avoid drying. Pollen was placed onto a papilla cell from the mounted anthers using a micromanipulator (MMO-4, Narishige) and photographed at 1-min intervals for 15 min following the initiation of pollination using a wide-field microscope (Axio Observer 7, Zeiss) with a 20 × objective (Zeiss).

After obtaining data, we used the pollen width as the indicator of pollen hydration. Pollen width was calculated by the “fit ellipse” function of Fiji software (Schindelin et al. 2012) as the minor axis length. Pollen hydration rate was defined as percentage changes in pollen width and estimated by the following equation:

2 $$\begin{array}{c}\frac{{L}_{t}-{L}_{0}}{{L}_{0}}\times 100\end{array}$$

where ${L}_{t}$ is the width of pollen at $t$ minutes after pollination and ${L}_{0}$ is the initial width of pollen (Wang et al. 2017).

### Pollen tube observation by aniline blue staining

Pistils were inserted into 1% agar and pollinated by hand to cover the whole of the papilla cells with pollen. At 2 h after pollination, the pistils were place in 1 M NaOH at 60 °C for 40 min, then stained with aniline blue solution (0.1% aniline blue, 0.1 M K_3_PO_4_) at room temperature (Wong et al. 1994). Images of the pollen tube stained with aniline blue solution were obtained using a fluorescence microscope (Axio Imager A2, Zeiss).

### Statistical analysis

All plots were created using seaborn v0.13.2 (Waskom 2021) or ggplot2 v3.5.1 (Wickham 2016). Dunnett’s test was performed using scipy v1.14.0 (Virtanen et al. 2020). The Tukey-HSD test and Welch’s *t* test were performed using pingouin v0.5.4 (Vallat 2018).

**Fig. 1:**
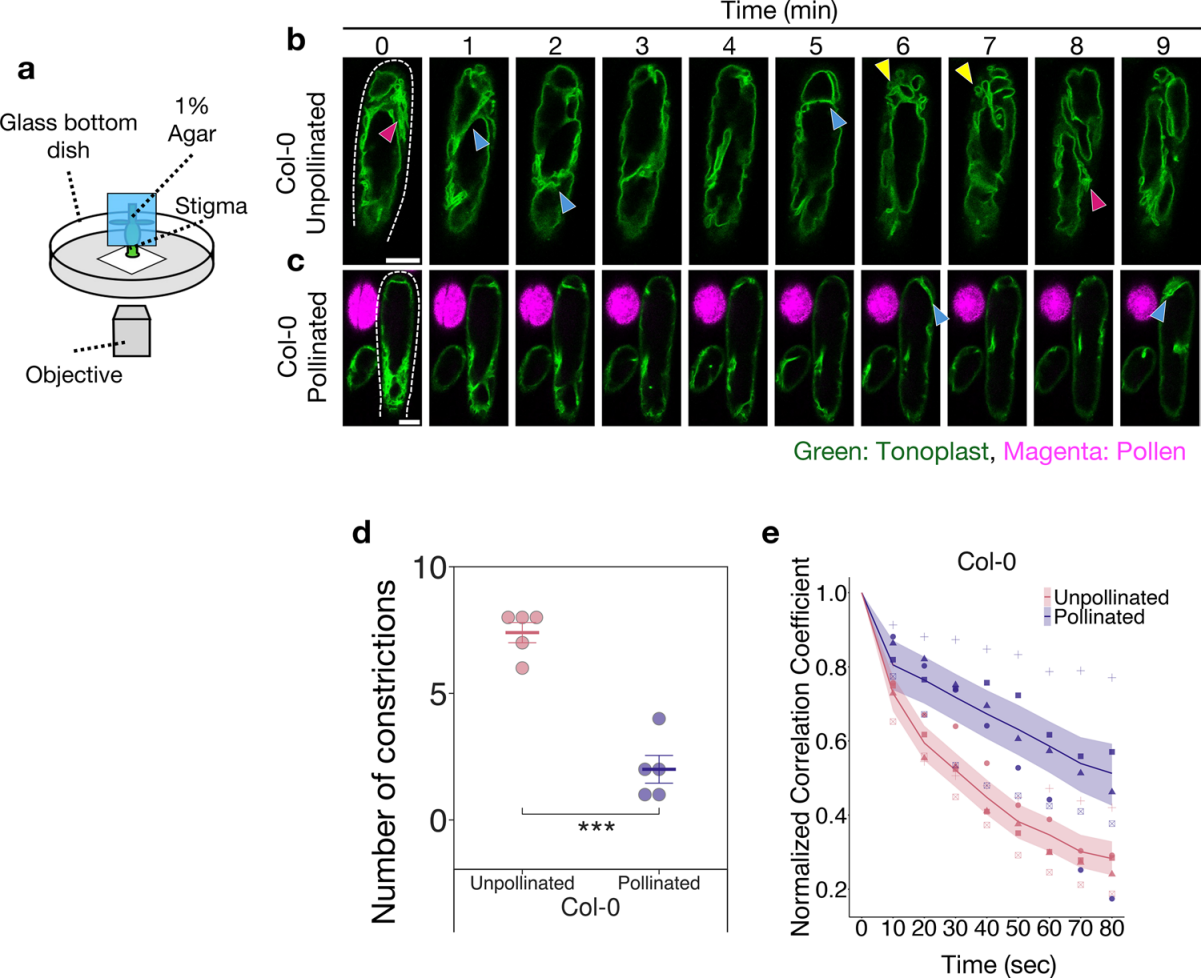
Live imaging of vacuoles in stigma papilla cells before and after pollination. **a** Illustration of sample setup for live-cell time-lapse imaging of papilla cells. Pistils were inserted into 1% agar blocks and mounted on glass-bottom dishes with the papilla cells placed in contact with the cover glass. **b**, **c** Representative image sequences of GFP fluorescence in the tonoplast of an unpollinated (**b**) and a pollinated (**c**) papilla cell in *35Spro-TTS::GFP* transgenic plants. Observation of pollinated papilla cells started at 3 min post-pollination. *UBQ10pro-PIP2a::mCherry*-expressing pollen of Col-0 background was applied to the papilla cells by hand pollination. In all images, the tip of the papilla cell is at the top of the image. White dotted lines indicate the outline of the papilla cells. Red arrowheads indicate tubular vacuoles formed by constriction of central vacuoles. Yellow arrowheads indicate small vacuoles formed by constriction of central vacuoles. Blue arrowheads indicate the site of constriction observed in central vacuoles. Scale bar: 10 µm. **d** Number of constrictions of the central vacuoles during 10 min (n = 5). Error bars indicate mean ± SEM (standard error of the mean). Statistical results were obtained using the Tukey-HSD test: ***p < 0.001. **e** Time variation of the normalized correlation coefficient with respect to the first image at 10-s intervals (n = 5). Each point represents the value at each point in time from each papilla cell. The same symbols indicate data from the same cell. Each line and shaded area indicate the median value and 95% credible interval of posterior distributions of the mean of the normalized correlation coefficient for each time period

**Fig. 2:**
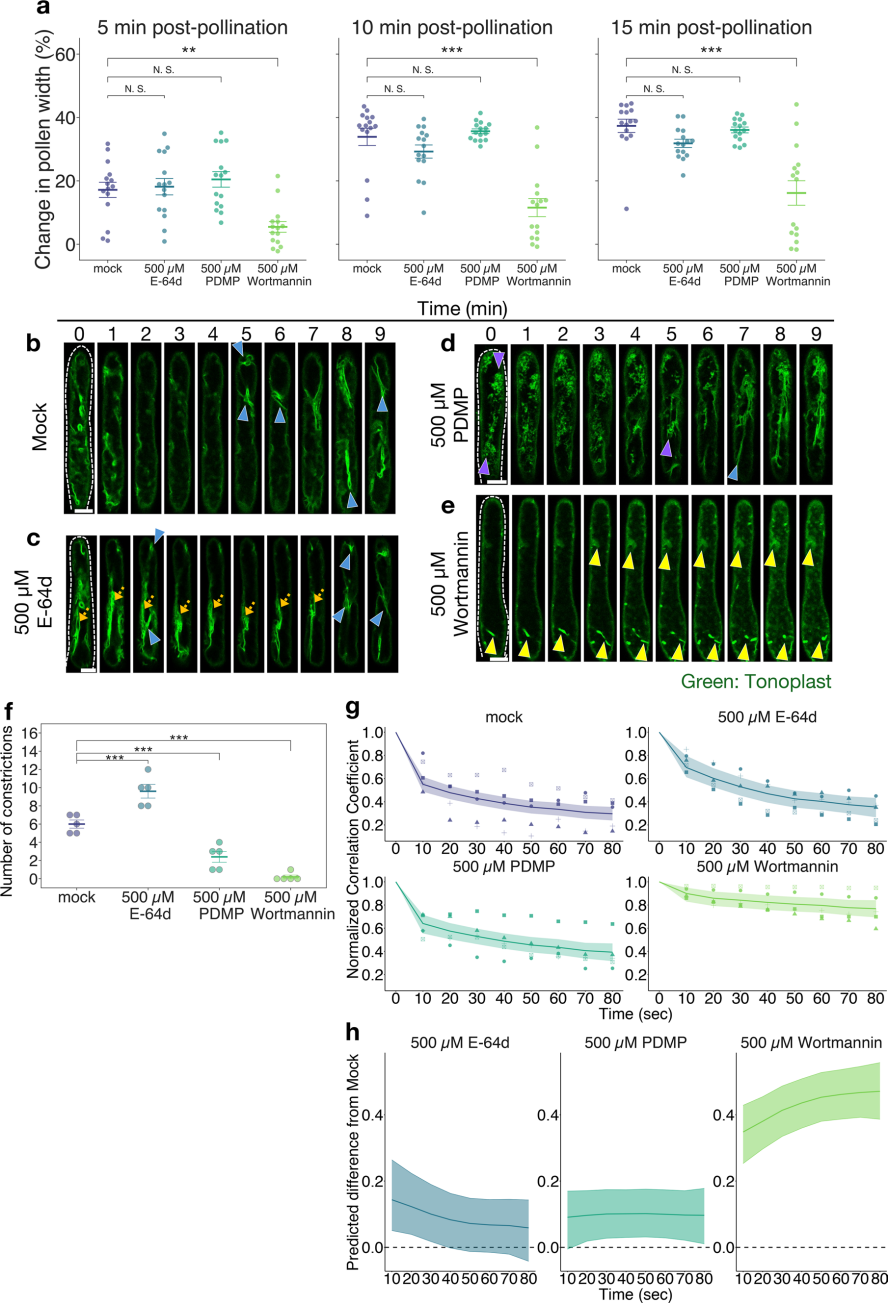
The effects of chemical treatment on vacuoles in stigma papilla cells. **a** Pollen hydration rate at 5 min, 10 min, and 15 min after pollination (n = 15). Error bars indicate mean ± SEM. **b**–**e** Representative image sequences of GFP fluorescence in tonoplast of unpollinated *35Spro-TTS::GFP* papilla cells treated with mock (**b**), 500 µM E-64d (**c**), 500 µM PDMP (**d**), and 500 µM wortmannin (**e**). Blue arrowheads indicate the site of constriction in central vacuoles. Orange arrows indicate the stacked tubular vacuoles. Purple arrowheads indicate small vacuoles taken up into central vacuoles in the papilla cells treated with PDMP. Yellow arrowheads indicate invagination of the tonoplast in the papilla cells treated with wortmannin. **f** Number of constrictions of the central vacuoles during 10 min (n = 5). Error bars indicate mean ± SEM. **g** The time variation of the normalized correlation coefficient with respect to the first image at 10-s intervals (n = 5). The data are presented as in Fig. 1e. **h** Time variation of the difference in normalized correlation coefficients with respect to mock treatment in (**g**). Each line and ribbon indicates the median value and 95% credible interval of posterior distributions of the difference for each time period. Statistical results in (**a**) and (**f**) were obtained by Dunnett’s test with the mock treatment as the control group: **p < 0.01; ***p < 0.001; N.S., not significant

For analyzing time series data of the normalized correlation coefficient (Figs. 1e, 2g, h, 4h, i, 5d, e), we used the state space model in the Bayesian inference (van de Schoot et al. 2021; Matsuura 2022). Means of the normalized correlation coefficient at the time variation and the differences between control and experimental groups were estimated using the model.

The system model is given by:

3 $$\begin{array}{c}\mu \left[t\right]\sim {\text{N}}{\text{o}}{\text{r}}{\text{m}}{\text{a}}{\text{l}}\left(\mu \left[t-1\right], {\sigma }_{\mu }\right),\\ t=2, \cdots ,T\end{array}$$

4 $$\begin{array}{c}{\text{delta}}\left[t\right]\sim {\text{Cauchy}}\left({\text{delta}}\left[t-1\right], {\sigma }_{\text{delta}}\right),\\ t=2,\cdots ,T\end{array}$$

where $t, T$ is time, $\mu [t]$ is the parameter for mean of the normalized correlation coefficient in the control group, ${\text{delta}}[t]$ is the parameter for difference of the normalized correlation coefficient from the control group, and ${\sigma }_{\mu }, {\sigma }_{\text{delta}}$ are parameters for standard deviation. Cauchy is the probability distribution given by

$$\text{Cauchy(}\theta |\mu ,\sigma \text{)}= \frac{1}{\pi \sigma }\frac{1}{1+{(\left(\theta -\mu \right)/\sigma )}^{2}}$$

The observation model is given by:

5 $$\begin{array}{c}{\text{NCC}}_{\text{control}}[t]\sim {\text{B}}{\text{e}}{\text{t}}{\text{a}} \, {\text{p}}{\text{r}}{\text{o}}{\text{p}}{\text{o}}{\text{r}}{\text{t}}{\text{i}}{\text{o}}{\text{n}}\left(\mu \left[t\right], \kappa \right),\\ t=1,\cdots ,T\end{array}$$

6 $$\begin{array}{c}{\text{NCC}}_{\text{experimental}}[t]\sim {\text{B}}{\text{e}}{\text{t}}{\text{a}} \, {\text{p}}{\text{r}}{\text{o}}{\text{p}}{\text{o}}{\text{r}}{\text{t}}{\text{i}}{\text{o}}{\text{n}}\left(\mu \left[t\right]+{\text{delta}}\left[t\right], \kappa \right), \\t=1,\cdots ,T\end{array}$$

where ${\text{NCC}}_{\text{control}}[t], {\text{NCC}}_{\text{experimental}}[t]$ are observed data of the normalized correlation coefficient, and $\kappa$ is the shape parameter of

$$\begin{aligned}&\text{Beta proportion}\left(\theta |\mu , \kappa \right)\\&=\frac{1}{B(\mu \kappa , (1-\mu )\kappa )}{\theta }^{\mu \kappa -1}{(1-\theta )}^{\left(1-\mu \right)\kappa -1}\end{aligned}$$

The parameters were estimated using Stan v2.35.0 (Stan Development Team 2024) and CmdStanR v0.8.1 (Gabry et al. 2024). A uniform distribution set by default by Stan was used for the prior distribution of all parameters.

## Results

### Tonoplast dynamics in papilla cells before and after pollination

We generated transgenic *Arabidopsis thaliana* plants expressing the tonoplast targeting sequence fused to GFP (*35Spro-TTS::GFP*) to visualize vacuole changes in papilla cells. As shown in Fig. 1a, live-cell time-lapse imaging of vacuoles in Col-0 papilla cells was performed using laser-scanning confocal microscopy by mounting pistils with the papilla cells in contact with a glass-bottom dish. In unpollinated papilla cells, continuous changes in the shape of the vacuoles, such as constriction and fusion, were observed during 10 min of imaging (Fig. 1b; Supplementary Video S1). In constriction sites of central vacuoles, actin bundles, which regulate vacuole morphology in papilla cells (Iwano et al. 2007; Scheuring et al. 2016), accumulated (Supplementary Fig. S1b). Despite these changes, the large vacuoles, which occupied most of the volume of papilla cells, remained in the center of the cells. Additionally, small and tubular vacuoles around the large central vacuole were observed (Fig. 1b, red and yellow arrowheads). The smaller vacuoles and tubular vacuoles emerged from the large central vacuole. They underwent a significant change in volume through a process of repeated fission and fusion, resulting in a substantial alteration in vacuolar morphology. Additionally, constriction of the central vacuole was observed (Fig. 1b, blue arrowheads). These observations suggest that the vacuoles in unpollinated papilla cells constrict and fuse repeatedly, resulting in large changes in their morphology.

Next, the vacuole behavior in pollinated papilla cells was observed, and compared to that of unpollinated ones (Fig. 1c; Supplementary Video S2). Pollen expressing *UBQ10pro-PIP2a::mCherry* was pollinated onto the papilla cells of *35Spro-TTS::GFP*-expressing pistils. Observation was initiated 3 min after pollination, followed by 10 min of imaging of vacuolar dynamics. In contrast to the unpollinated papilla cells, vacuole constriction and emergence of small and tubular vacuoles were markedly reduced, and a large central vacuole continued to occupy the most of the papilla cell (Fig. 1c, blue arrowheads; Supplementary Video S2). Indeed, when the frequency of constrictions of the central vacuoles was counted as the number of times a new vacuolar boundary appeared, it was found to be significantly lower after pollination than before (Fig. 1d). Furthermore, we quantified the spatiotemporal changes of vacuoles in the papilla cells using the normalized correlation coefficient. To quantify the variation in vacuole morphology, the time variation of the correlation coefficient from the reference state, defined as the vacuolar morphology at the start of observation (0 s), was calculated every 10 s, and the state space model was applied in the Bayesian inference to estimate the mean (Fig. 1e, see Materials and Methods section). This indicated that the temporal variation in vacuole morphology decreased after pollination compared to before pollination. These results suggest that the vacuole morphology of papilla cells before pollination continued to change due to frequent vacuolar constriction; however, after pollination, fusion from smaller vacuoles to larger ones occurred, and vacuolar constriction was reduced, resulting in smaller variations in vacuolar morphology.

### Effects of chemical treatment on vacuole constriction and morphological changes in papilla cells

To investigate the relationship between vacuole morphology and pollen hydration, we next examined pollen hydration when vacuole dynamics were altered by chemical treatments of *A. thaliana* papilla cells. In this experiment, we treated papilla cells with chemicals that have been demonstrated in previous research to affect vacuole dynamics: E-64d (an inhibitor of vacuole fusion; Gao et al. 2005), PDMP (an inducer of the fusion of small vacuoles into large vacuoles; Krüger et al. 2013), and wortmannin (an inducer of the fusion of vacuoles; Zheng et al. 2014; Alvarez et al. 2016), and measured the pollen hydration rate. When the pollen hydration rate was measured at three time points (5, 10, and 15 min after pollination) it was found to be significantly suppressed only in the wortmannin treatment, and this occurred at all time points (Fig. 2a).

Next, we observed vacuole dynamics in the unpollinated papilla cells treated with the chemicals (Fig. 2b–e). The vacuoles in the mock-treated unpollinated papilla cells exhibited the same behavior as in untreated papilla cells (Figs. 1b, 2b; Supplementary Video S3), including constriction of central vacuoles (blue arrowheads). In the E-64d treatment, vacuole constriction was frequent and the generated tubular vacuoles stacked around the central vacuoles (Fig. 2c, orange arrows; Supplementary Video S4). In the PDMP treatment, the central vacuoles showed membrane invagination and fusion with small vacuoles occurred (Fig. 2d, purple arrowheads; Supplementary Video S5). However, constriction of vacuoles was rarely observed (blue arrowheads). In contrast to E-64d and PDMP, wortmannin treatment induced a large central vacuole and partial membrane invagination, and no vacuole constriction occurred (Fig. 2e; yellow arrowheads; Supplementary Video S6). The effects of these chemicals on vacuole dynamics were consistent with previous reports (Gao et al. 2005; Krüger et al. 2013; Zheng et al. 2014; Alvarez et al. 2016), and vacuoles in pollinated papilla cells treated with these chemicals showed similar phenotypes to that before pollination (Supplementary Fig. S2; E-64d, PDMP, 500 µM wortmannin).

Furthermore, the frequency of vacuole constriction increased with E-64d treatment, and decreased with PDMP and wortmannin treatments compared to mock treatment (Fig. 2f). The time variation of the normalized correlation coefficient was calculated (Fig. 2g). We estimated the difference in the normalized correlation coefficient between the mock and chemical treatments at each time point by Bayesian inference (Fig. 2h, see Materials and Method section). From these results, we found that wortmannin treatment caused a large difference in changes of vacuole morphology (Fig. 2g, h). These results suggest that suppression of pollen hydration by wortmannin is partly related to the absence of vacuole morphological changes in papilla cells.

**Fig. 3:**
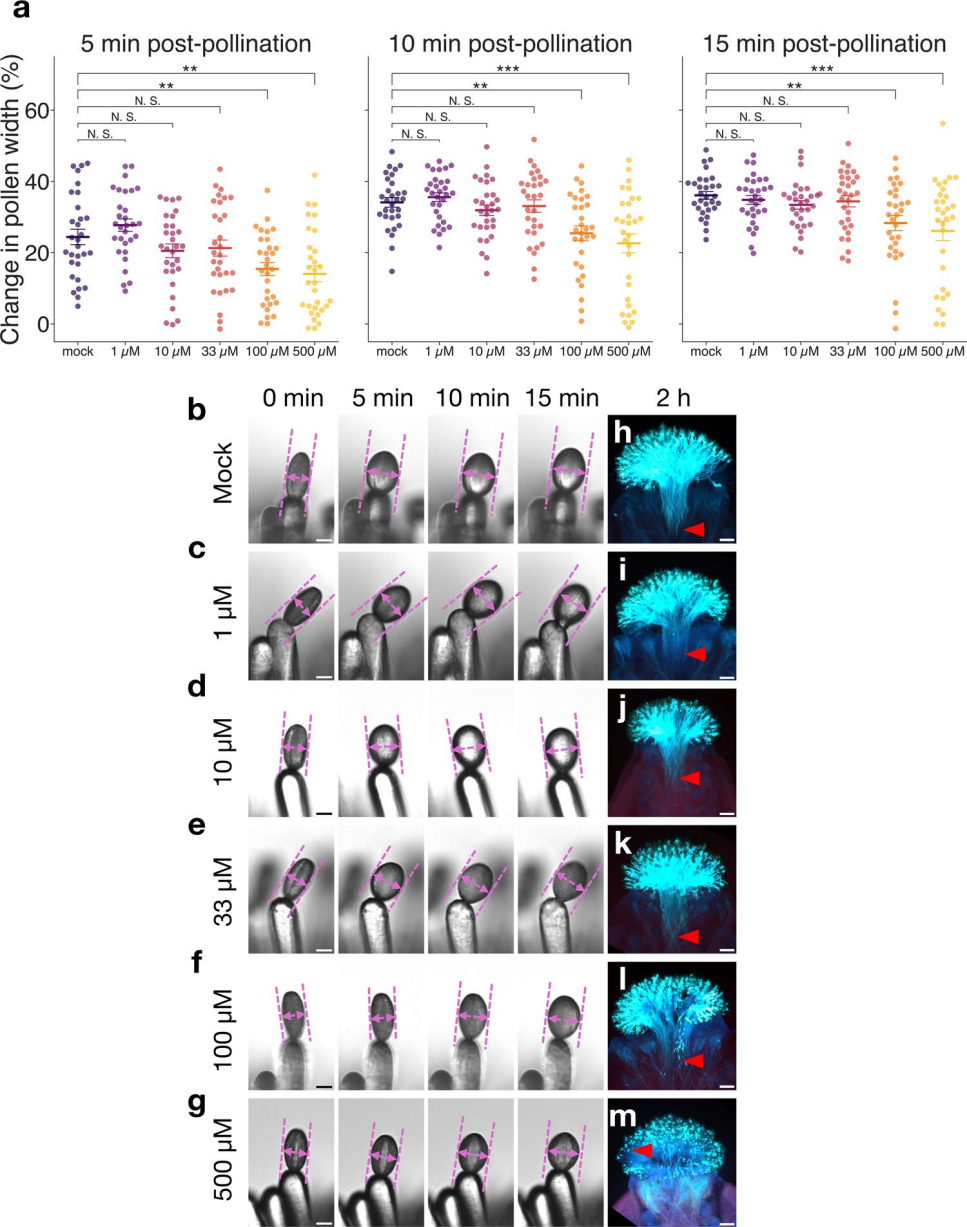
The effect of wortmannin treatment on pollen hydration. **a** Pollen hydration rate at 5 min, 10 min, and 15 min after pollination in the mock and each wortmannin treatment (n = 30). The data are presented as in Fig. 2a. Statistical results were obtained by Dunnett’s test with the mock treatment as the control group: **p < 0.01; ***p < 0.001; N.S., not significant. **b**–**g** Images of pollen hydration on the papilla cells treated with mock (**b**), 1 µM (**c**), 10 µM (**d**), 33 µM (**e**), 100 µM (**f**), and 500 µM (**g**) wortmannin at 5 min, 10 min, and 15 min after pollination. Pink dashed lines show measurements of pollen width. Scale bar: 10 µm. **h**–**m** Images of aniline blue staining of pollen tubes in the stigmas treated with mock (**h**), 1 µM (**i**), 10 µM (**j**), 33 µM (**k**), 100 µM (**l**), and 500 µM (**m**) wortmannin. Samples were stained at 2 h after pollination. Red arrowheads indicate the representative length of pollen tubes. Scale bar: 100 µm

**Fig. 4:**
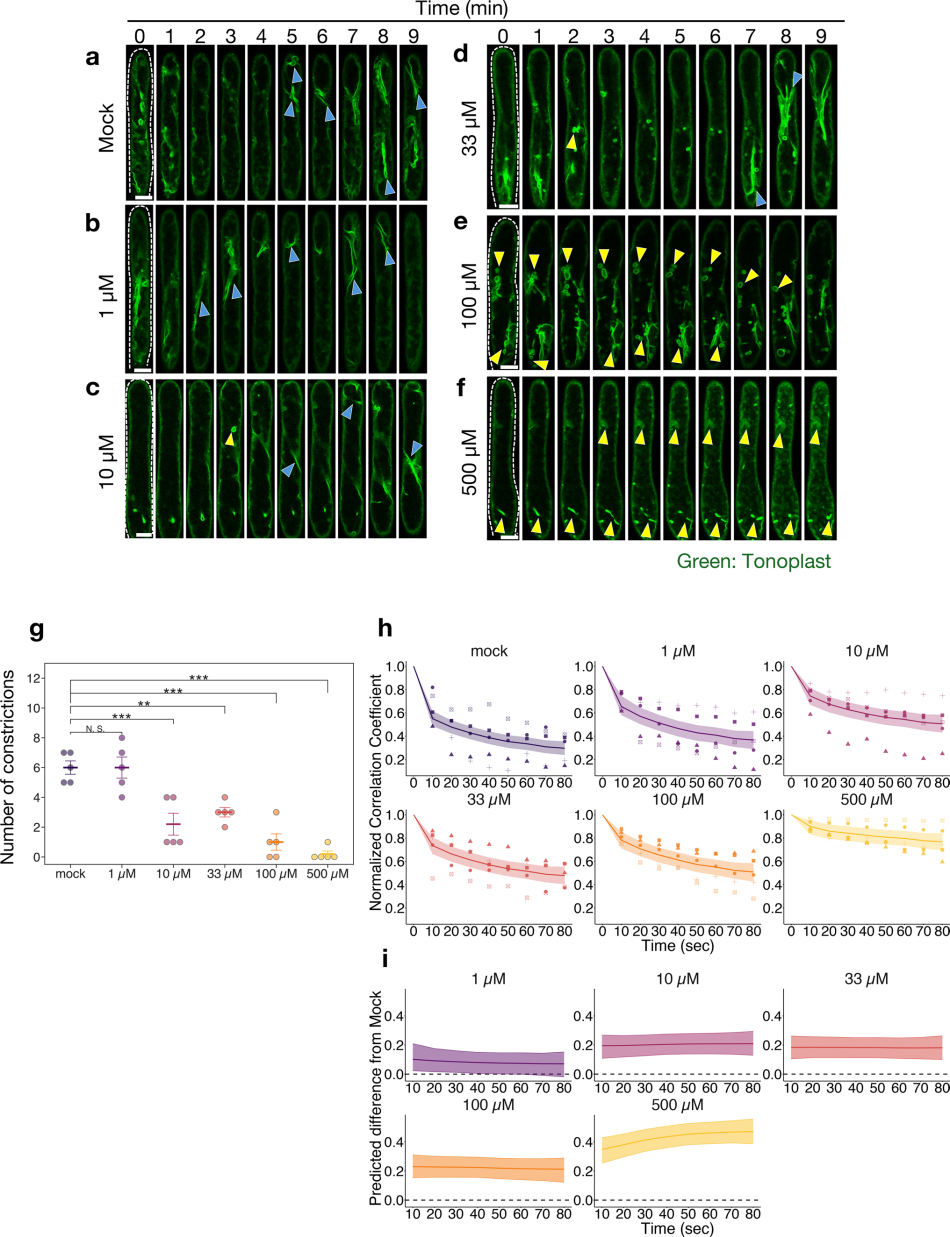
The effects of wortmannin treatment on the vacuole dynamics of stigma papilla cells. **a**–**f** Representative image sequences of GFP fluorescence in the tonoplast of unpollinated papilla cells treated with mock (**a**), 1 µM (**b**), 10 µM (**c**), 33 µM (**d**), 100 µM (**e**), and 500 µM (**f**) wortmannin in *35Spro-TTS::GFP* transgenic plants. In all images, the tip of the papilla cell is at the top of the image. White dotted lines indicate the boundaries of the papilla cells. Blue arrowheads indicate the site of constriction observed in central vacuoles. Yellow arrowheads indicate the invagination of the tonoplast in the papilla cells treated with wortmannin. Scale bar: 10 µm. **g** Number of constrictions of the central vacuoles in unpollinated papilla cells treated with wortmannin for 10 min (n = 5). Error bars indicate mean ± SEM. Statistical results were obtained using Dunnett’s test with the mock treatment as the control group: **p < 0.01; ***p < 0.001; N.S., not significant. **h** The time variation of the normalized correlation coefficient with respect to the first image at 10-s intervals in the unpollinated papilla cells treated with wortmannin (n = 5). The data are presented as in Fig. 1e. **i** Time variation of the difference in normalized correlation coefficients with respect to mock treatment in (**h**). The data are presented as in Fig. 2h

To investigate in detail the inhibition of vacuole dynamics and pollen hydration by wortmannin treatment, the concentration-dependent effects of wortmannin on pollen hydration and vacuole dynamics in unpollinated papilla cells were next examined (Figs. 3, 4). Treatment with 1–500 µM wortmannin gradually inhibited pollen hydration; in particular, hydration of pollen on 100 µM and 500 µM wortmannin-treated papilla cells was significantly lower than that on mock-treated papilla cells at 5, 10, and 15 min after pollination (Fig. 3a–g). In addition, pollen tube elongation in the pistils at 2 h after pollination was inhibited by 500 µM wortmannin treatment but not by mock, 1 µM, 10 µM, 33 µM, and 100 µM wortmannin treatments (Fig. 3h–m). Next, we observed the vacuoles in unpollinated and pollinated papilla cells treated with the same concentrations of wortmannin as used in the measurements of pollen hydration rate (Fig. 4a–f). The frequency of vacuole constrictions with 1 µM wortmannin treatment was similar to that of the mock treatment; however, reduction in vacuole constriction was observed at higher concentrations (Fig. 4a–g, blue arrowheads; Supplementary Videos S7–S10). Additionally, abnormal vacuole structures appeared in the unpollinated papilla cells treated with 10, 33, 100, and 500 µM wortmannin (Fig. 4c–f, yellow arrowheads). In particular, these abnormal structures were frequently observed in unpollinated papilla cells treated with 100 and 500 µM wortmannin, which suppressed pollen hydration (Fig. 4e, f). When the variation in vacuole morphology of the unpollinated cells was compared between the wortmannin and mock treatments, the differences were observable at concentrations of 10 µM and higher, as vacuole constriction was reduced (Fig. 4h, i). In particular, it was shown that only the difference between 500 µM wortmannin and mock treatment increased over time (Fig. 4i). These results indicated that wortmannin caused the suppression of pollen hydration, and a lack of vacuole constriction and vacuole morphological changes in the papilla cells in a concentration-dependent manner. It is suggested that morphological changes and constriction of vacuoles are related to the regulation of pollen hydration.

### Vacuole dynamics in papilla cells of *rop2* mutant

**Fig. 5:**
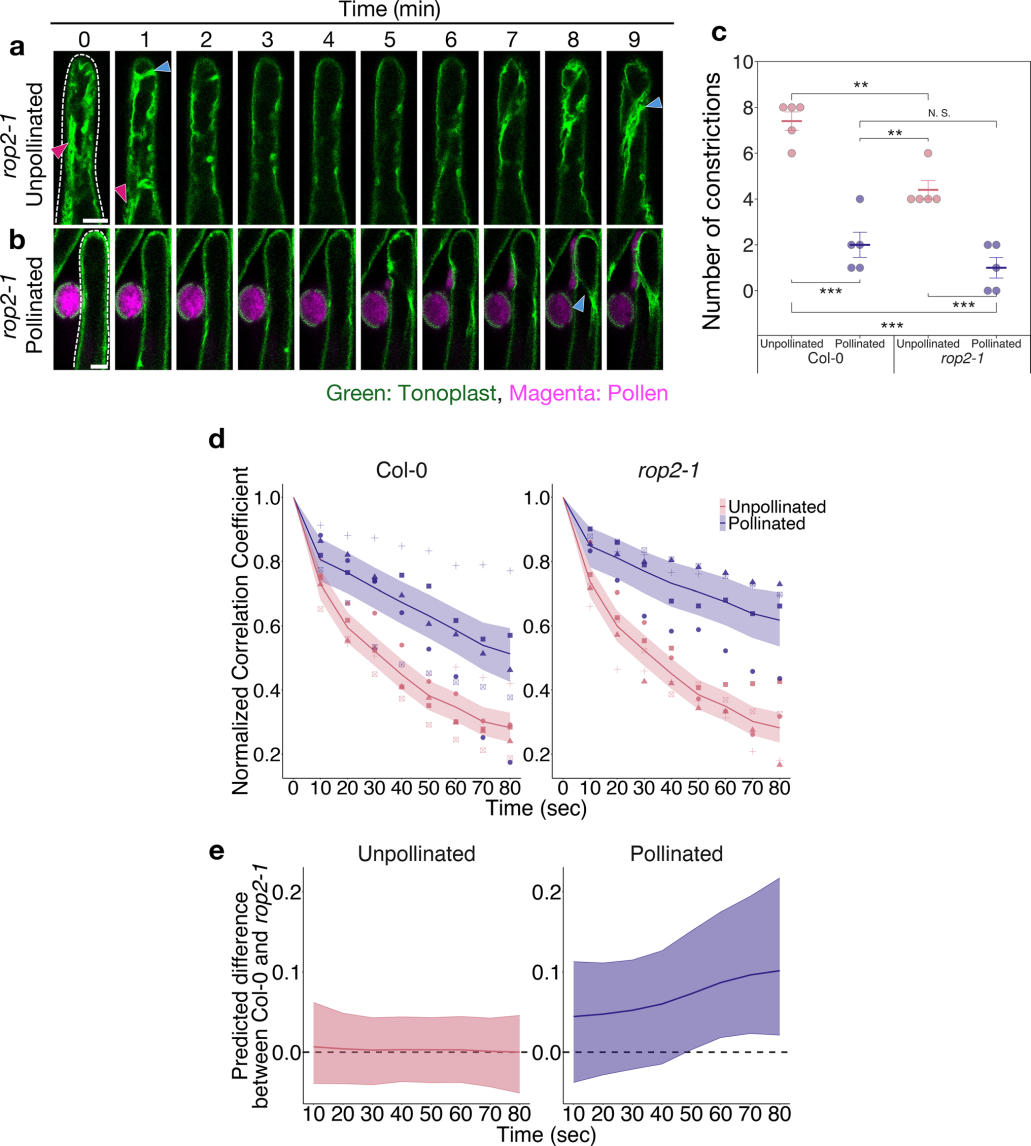
The vacuole dynamics in the *rop2-1* mutant. **a**, **b** Representative image sequences of GFP fluorescence in the tonoplast of an unpollinated (**a**) and a pollinated (**b**) papilla cell expressed by *AtS1pro-TTS::GFP* in the *rop2-1* background. Observation of pollinated papilla cells started at 3 min post-pollination. Red arrowheads indicate tubular vacuoles. Blue arrowheads indicate the site of constriction observed in central vacuoles. **c** Number of constrictions of the central vacuoles in papilla cells of Col-0 and *rop2-1* (n = 5). Error bars indicate mean ± SEM. Statistical results were obtained using the Tukey-HSD test: **p < 0.01; ***p < 0.001; N.S., not significant. **d** The time variation of the normalized correlation coefficient with respect to the first image at 10-s intervals in papilla cells of Col-0 and *rop2-1* (n = 5). The data are presented as in Fig. 1e. **e** Time variation of the difference in normalized correlation coefficients between Col-0 and *rop2-1* in (**d**). The data are presented as in Fig. 2h

To further investigate the role of vacuoles in transferring water from papilla cells to pollen, we examined vacuole dynamics in plants with enhance pollen hydration, as opposed to the wortmannin treatment that inhibited pollen hydration and vacuole dynamics. Rho-like GTPase from plants 2 (ROP2) is a negative regulator of compatible pollen hydration, and its mutant displays a higher pollen hydration rate (Liu et al. 2021). We confirmed the increased pollen hydration rate on papilla cells in the *rop2-1* mutant (Jeon et al. 2008; Liu et al. 2021; Supplementary Fig. S3). We then generated transgenic plants expressing *AtS1pro-TTS::GFP* in the *rop2-1* mutant background and observed vacuole dynamics in their papilla cells (Fig. 5a, b; Supplementary Videos S11, S12). Vacuole constriction in the *rop2-1* mutant was observed before pollination, although few tubular vacuoles were seen, as observed in Col-0 (Fig. 1b), and the central vacuoles remained largely intact due to the reduction of constriction (Fig. 5a, c, blue and red arrowheads). After pollination in the *rop2-1* mutant, there was a central, large vacuole in the papilla cells (Fig. 5b). The frequency of vacuole constriction in the *rop2-1* mutant was lower than before pollination (Fig. 5b, c, blue arrowheads). Next, the alterations in vacuole morphology, as shown by the normalized correlation coefficient, were compared between the *rop2-1* mutant and Col-0 (Fig. 5d, e). Before pollination, changes in vacuole morphology in Col-0 and the *rop2-1* mutant were the same (Fig. 5d, e; unpollinated). However, after pollination, Col-0 and the *rop2-1* mutant showed differences in the changes in vacuole morphology. In particular, after 50 s, the *rop2-1* mutant exhibited a clear difference from Col-0, that is, there was less change in vacuolar morphology (Fig. 5d, e; pollinated). These results suggest that a less variable morphology of vacuoles observed in the *rop2-1* pollinated papilla cells may be linked to a faster pollen hydration rate.

## Discussion

In this study, we observed the vacuoles in *A. thaliana* papilla cells before and after pollination using live-cell time-lapse imaging. In unpollinated papilla cells, the vacuoles, which continuously changed their shapes by fusing and constricting, were observed (Fig. 1b). There have been no previous report of plant cells showing such a high frequency of vacuolar morphological changes without changes in cell volume. Thus, the change in the shape of the vacuoles in the unpollinated papilla cells might be a specific phenomenon in papilla cells. After pollination, the small vacuoles were no longer observed, and a single vacuole was maintained (Fig. 1c). The central vacuole is hypothesized to be a fusion of the small and tubular vacuoles. In ultra-high-voltage electron microscopy (ultra-HVEM) observation of the papilla cells of *B. rapa* before pollination, small and tubular vacuoles were abundant near the apical cell membrane of the papilla cells, while central vacuoles were present at the basal side of the papilla cells (Iwano et al. 2007). Furthermore, the central vacuole moved from the basal side of the papilla cell to near the pollen attachment site after pollination, while the small and tubular vacuoles at the apical side of the papilla cell were reduced (Iwano et al. 2007). Due to the utilization of freeze-fixed tissue sections in ultra-HVEM observations, it is difficult to capture the dynamic movement of vacuolar morphology before and after pollination. The repeated constriction and fusion of vacuoles within the papilla cells captured in the live imaging in our study suggests that small and tubular vacuoles, often observed in *B. rapa* papilla cells before pollination, fuse with central vacuoles after pollination. Furthermore, reduced vacuolar constriction and morphological changes compared to the wild type were found in unpollinated papilla cells of *rop2* mutants, which are known to have an increased pollen hydration rate (Jeon et al. 2008; Liu et al. 2021; Fig. 5; Supplementary Fig. S3). This indicates that the maintenance of one large central vacuole may be essential for water supply to pollen. Therefore, papilla cells of the *rop2* mutant, in which one large central vacuole was formed before pollination, were primed ready to supply water even before pollen attachment, which may have increased the pollen hydration rate after pollination.

Regulation by the cytoskeleton and cytoplasmic streaming have been suggested as factors that alter vacuole morphology in plant cells. However, the detailed mechanisms of these controls remain unclear (Zirkle 1937; Cui et al. 2020; Takatsuka et al. 2023). Treatment of *B. rapa* papilla cells with cytochalasin D, an actin polymerization inhibitor, inhibits pollen hydration (Iwano et al. 2007). With this treatment, the actin skeleton collapses within the papilla cells, and spherical vacuoles are observed to be assembled on the surface of the central vacuole (Iwano et al. 2007). Thus, in *B. rapa* papilla cells, regulation of actin is considered to result in vacuole morphological changes and regulate pollen hydration. Therefore, it is likely that actin causes vacuolar morphological changes in *A. thaliana,* as observed in this study.

Water transport across the plasma membrane of plant cells is a passive process, which takes place by osmotic pressure differences between intracellular and extracellular membranes. Cell elongation, stomatal opening and closing, and leaf movement in legumes are induced by changes in intracellular and extracellular osmotic pressure that allow water to move in and out of the cell, resulting in changes in cell volume (Cosgrove 2000; Wang et al. 2001; Shimazaki et al. 2007; Mano and Hasebe 2021). Of these responses, guard cells, which form stomata, have been extensively investigated in relation to vacuolar morphology and intracellular water retention. Guard cells induce stomata closure by reducing cell volume by draining intracellular water, and conversely induce stomata opening by increasing cell volume by drawing water into the cell (Roelfsema and Hedrich 2005; Shimazaki et al. 2007). It has been shown that during stomatal closure, the vacuole exists as many small vacuoles, but during opening it becomes one central vacuole, thereby increasing cell volume (Gao et al. 2005; Andrés et al. 2014; Mirasole et al. 2023). In guard cells, changes in intracellular osmotic pressure determine the direction of water flow in and out of the cells. As observed in this study, the frequent constriction of vacuoles in papilla cells before pollination suggests that the intracellular osmotic pressure is low, similar to the osmotic pressure regulation in guard cells. In pollinated papilla cells, a distinct central vacuole formed (Fig. 1c), suggesting that the osmotic pressure in the papilla cells after pollination is higher than before pollination. This vacuole fusion would be necessary for keeping enough water in papilla cells, which is transferred to pollen according to the difference in osmolarity between papilla cells and pollen. The increased osmotic pressure in papilla cells by pollination may play a role in drawing water into vacuoles. To our knowledge, the mechanism of water extrusion from vacuoles in papilla cells has not been elucidated. This is also a challenge for the future.

Wortmannin treatment of papilla cells had a different effect on pollen hydration from vacuole fusions in wild-type pollinated papilla cells. Wortmannin treatment suppressed vacuole constriction (Fig. 4). However, pollen hydration was also suppressed, although it appeared similar to the vacuole morphology in wild-pollinated papilla cells (Figs. 1c, d, 2a, e, 3, 4a-f; Supplementary Fig. S2a, d–h). In guard cells, wortmannin treatment, which inhibits phosphatidylinositol 3-kinase (PI3K), induces vacuole fusion by PtdIns(3)P depletion and small stomatal opening, which is associated with increased intracellular osmotic pressure (Zheng et al. 2014). This suggests that fusion of the small and tubular vacuoles into the central vacuoles in wortmannin-treated papilla cells is also caused by PtdIns(3)P depletion. This effect may be involved in the inhibition of pollen hydration caused by PI3K inhibition of wortmannin, which may be associated with increased intracellular osmotic pressure. Therefore, it is conceivable that the osmotic pressure in pollinated papilla cells would be extremely higher than that in wortmannin-treated papilla cells, inhibiting pollen hydration (Supplementary Fig. S4). In papilla cells treated with 500 µM wortmannin, in addition to the influx of water into the inner part of the vacuole due to increased osmolarity, vacuole morphology did not change, and invagination of the tonoplast became more pronounced, which also would be associated to increase intracellular osmolarity (Figs. 2, 3). Effects of chemical treatments on vacuole morphology were also observed in papilla cells treated with E-64d and PDMP, but these did not affect pollen hydration (Fig. 2). E-64d is known to inhibit vacuole fusion in guard cells and suppress stomatal opening (Gao et al. 2005). In papilla cells, E-64d increased the number of vacuole constrictions, which may lead to the generation of tubular vacuoles stacked around central vacuoles (Fig. 2c, f). This effect is likely due to the inhibition of vacuole re-fusion by E-64d. E-64d treatment results in a low intracellular osmotic pressure, which facilitates the movement of water outwards (Gao et al. 2005). PDMP increase intracellular ceramide concentrations, and PDMP-treated *A. thaliana* roots have fused vacuoles (Krüger et al. 2013). In papilla cells, PDMP may reduce the number of vacuoles by fusing small and tubular vacuoles into a central vacuole, which decreases the number of vacuole constrictions (Fig. 2f). Although the effect of PDMP on guard cells has not been reported, sphingosine-1-phosphate (S1P) is known to promote stomatal closure (Coursol et al. 2003). Given that the PDMP treatment in this study is similar to the action of S1P on guard cells, the treatment may reduce intracellular osmotic pressure and facilitate the movement of water to the outside. This suggests that E-64d and PDMP altered the vacuolar morphology of the papilla cells, but caused a reduction in intracellular osmotic pressure, so that pollen hydration occurred normally (Supplementary Fig. S4). Although central vacuoles were formed in papilla cells both during wortmannin treatment and after pollination, invagination of the tonoplast was constantly observed only with wortmannin treatment. This suggests that a function of the central vacuole is reduced by the tonoplast invagination, which may have inhibited pollen hydration.

Pollen hydration is thought to occur according to the osmotic pressure difference between pollen and papilla cells (Roberts et al. 1984; Dickinson 1995). Immediately after pollination, dry pollen is under very high osmotic pressure (low water potential). This suggests that the pollinated papilla cells that formed a central vacuole have a lower osmotic pressure (higher water potential) than pollen. Therefore, it can be assumed that the central vacuole is the source of water supply to pollen in pollinated papilla cells.

Wortmannin is also known to effect vesicular transport into the vacuole and between the *trans*-Golgi network and multivesicular bodies, affecting the subcellular localization of protein (Matsuoka et al. 1995; daSilva et al. 2005; Takáč et al. 2012). It is therefore possible that in papilla cells treated with wortmannin, water transport may have been inhibited, in addition to changes in vacuolar morphology and osmolarity, due to mislocalization of channels and transporters on the plasma membrane, including plasma membrane intrinsic proteins (PIPs) (Windari et al. 2021; Liu et al. 2025) and on the tonoplast, including tonoplast intrinsic proteins (TIPs). Therefore, we hypothesize that pollen hydration requires osmotic regulation through changes in vacuole morphology in the papilla cells, as well as membrane channels and transporters involved in water transport. Based on these results, we conclude that *A. thaliana* papilla cells control water transport to pollen by vacuole morphological changes and that osmotic regulation may be involved in this control.

## Supplementary Information

Below is the link to the electronic supplementary material.

Supplementary file1 (PDF 2752 KB)

Supplementary file2 (AVI 844 KB)

Supplementary file3 (AVI 1077 KB)

Supplementary file4 (AVI 618 KB)

Supplementary file5 (AVI 651 KB)

Supplementary file6 (AVI 779 KB)

Supplementary file7 (AVI 664 KB)

Supplementary file8 (AVI 544 KB)

Supplementary file9 (AVI 672 KB)

Supplementary file10 (AVI 667 KB)

Supplementary file11 (AVI 631 KB)

Supplementary file12 (AVI 745 KB)

Supplementary file13 (AVI 971 KB)

Supplementary file14 (docx 14 KB)

## Data Availability

All data supporting the findings of this study are available within the paper and the supplementary materials.
