# Supplementary figures and images for: The regulation of vacuole morphology in stigma papilla cells is involved in water transfer to pollen in Arabidopsis thaliana
Source: Plant Reprod. 2025 Jun 6;38(2):15. doi: 10.1007/s00497-025-00525-1 (PMC12144065; doi:10.1007/s00497-025-00525-1)

Supplementary  
Fig. S1

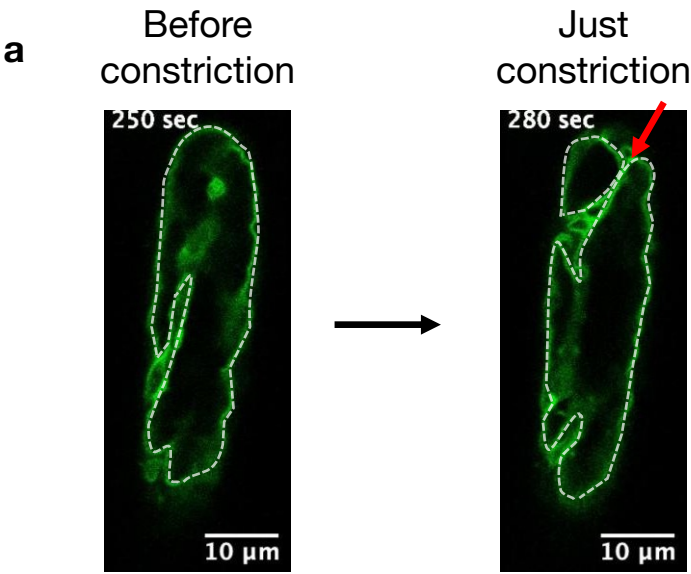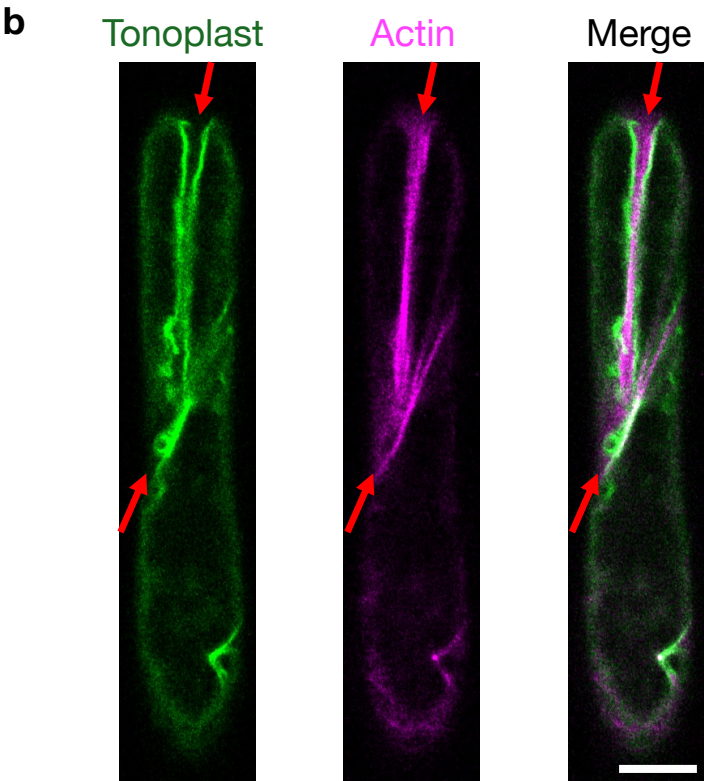

# Supplementary Fig. S2

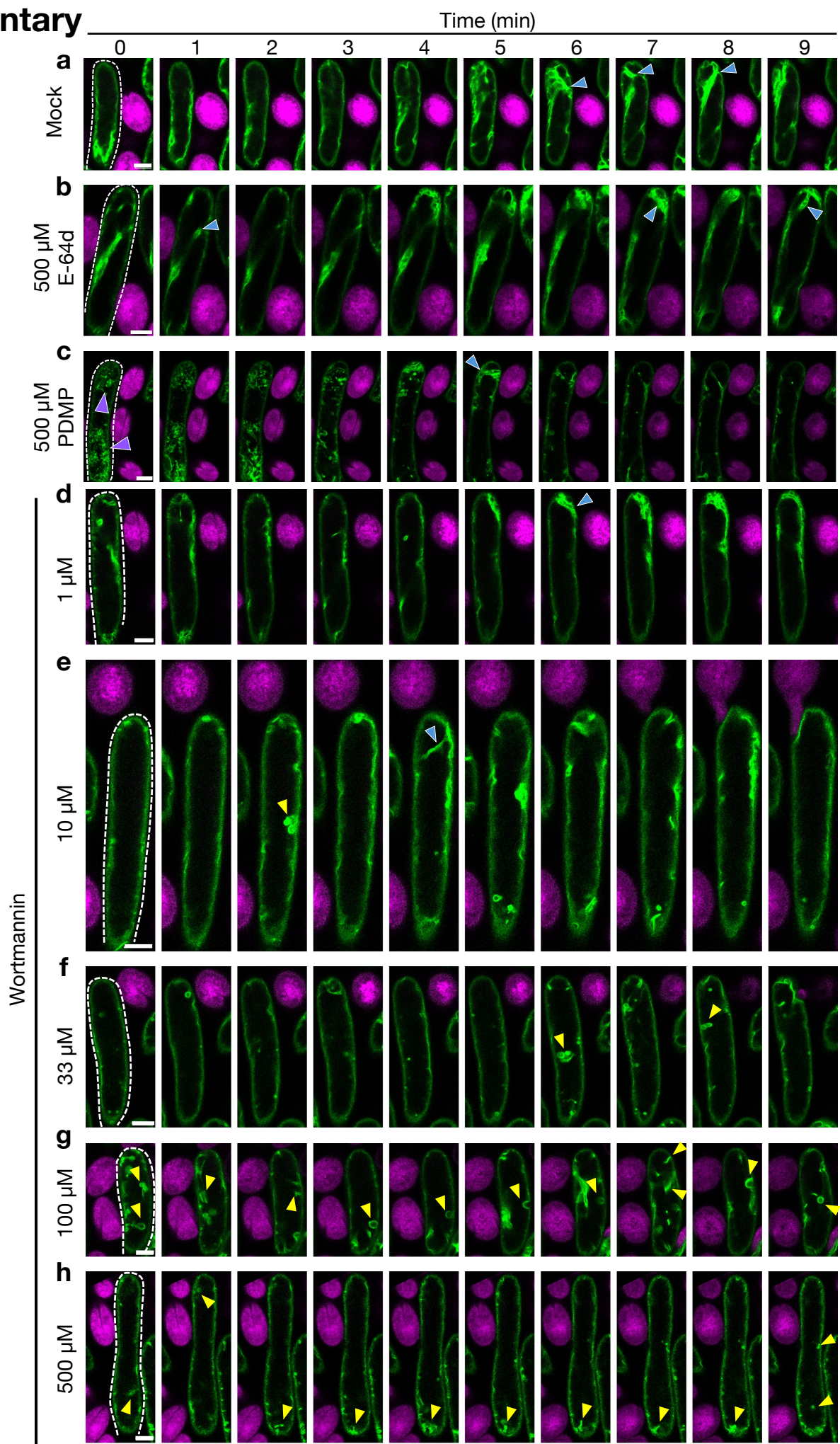

Supplementary  
Fig. S3

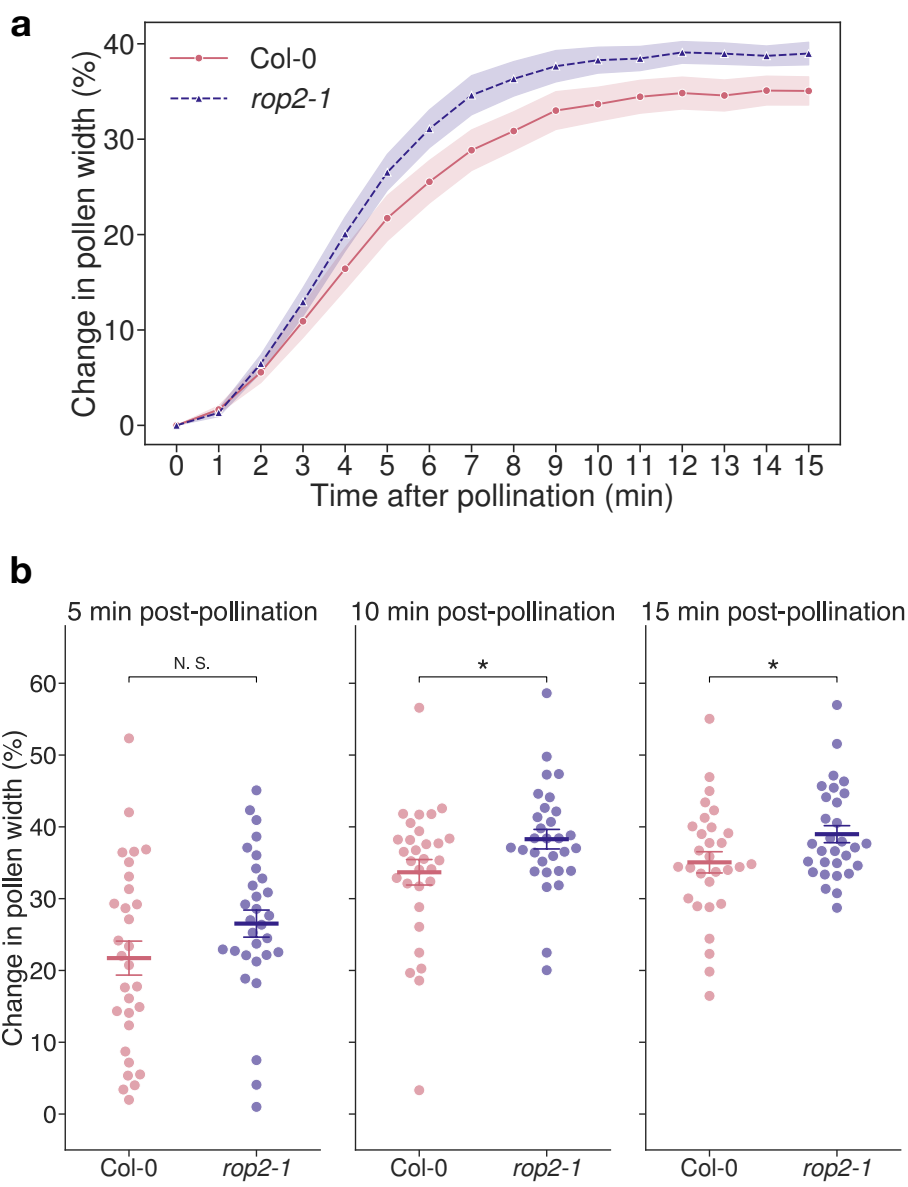

Supplementary  
Fig. S4

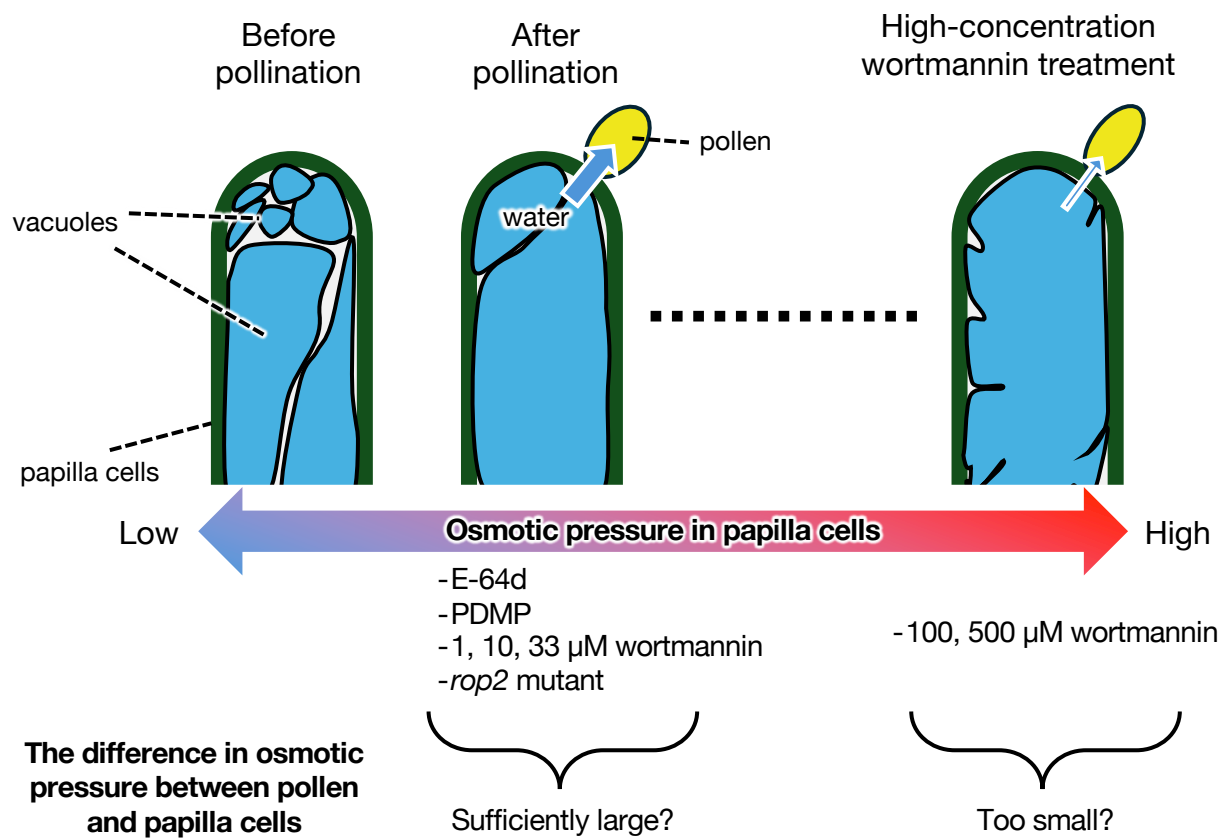

Supplement: Supplementary file 1 — Supplementary file1 (PDF 2752 KB) [file 497_2025_525_MOESM1_ESM.pdf]
