# Supplementary material for: The regulation of vacuole morphology in stigma papilla cells is involved in water transfer to pollen in Arabidopsis thaliana
Source: Plant Reprod. 2025 Jun 6;38(2):15. doi: 10.1007/s00497-025-00525-1 (PMC12144065; doi:10.1007/s00497-025-00525-1)
Supplement: Supplementary file 14 — Supplementary file14 (docx 14 KB) [file 497_2025_525_MOESM14_ESM.docx]

**Supplementary data**

**Supplementary Fig. S1** Vacuole constrictions in unpollinated papilla cells. (a) Representative images of the appearance of vacuole borders completely across papilla cells in Supplementary Video S1. White dotted lines trace the tonoplast signal of the central vacuole. Red arrows indicate the appearance of a vacuole border in the central vacuole. (b) Actin bundles accumulated at the vacuole borders in unpollinated papilla cells. Red arrows indicate the accumulated actin bundles at the vacuole border. Scale bar: 10 µm

**Supplementary Fig. S2** The effect of chemical treatments on vacuoles of pollinated papilla cells. Representative image sequences of the GFP fluorescence in tonoplast of unpollinated *35Spro-TTS::GFP* papilla cells from mock (a), 500 µM E-64d (b), 500 µM PDMP (c), and wortmannin 1 µM (d), 10 µM (e), 33 µM (f), 100 µM (g), and 500 µM (h). Observation of pollinated papilla cells started at 3 min post-pollination. *UBQ10-PIP2a::mCherry*-expressing pollen of Col-0 background was applied to the papilla cells by hand pollination. In all images, the tip of the papilla cell is at the top of the image. Blue arrowheads indicate the site of constriction observed in central vacuoles. Purple arrowheads indicate small vacuoles taken up into central vacuoles in the papilla cells treated with PDMP. Yellow arrowheads indicate invagination of the tonoplast in the papilla cells treated with wortmannin. Scale bar: 10 µm

**Supplementary Fig. S3** Pollen hydration on the *rop2-1* mutant papilla cells. (a) Pollen hydration rate on the papilla cells of Col-0 and *rop2-1* mutant during 15 min after pollination (n=30). The shaded area indicate mean ± SEM. (b) Pollen hydration on the papilla cells in Col-0 and *rop2-1* mutant at 5 min, 10 min, and 15 min after pollination. The symbols indicate the value for each pollen grain. Error bars indicate mean ± SEM. The statistical results were obtained using Welch’s *t* test: *, *p*<0.05; N.S., not significant.

**Supplementary Fig. S4** Hypothetical model proposing that osmotic pressure in papilla cells regulates pollen hydration. It is conceivable that low osmotic pressure in papilla cells would be sufficient for pollen hydration, while the extremely high osmotic pressure caused by wortmannin in papilla cells would inhibit water transfer from papilla cells to pollen. There may be an optimal range of osmotic pressure in papilla cells.

**Supplementary Video S1** The vacuoles in unpollinated papilla cells of a *35Spro-TTS-GFP* transgenic plant

**Supplementary Video S2** The vacuoles in pollinated papilla cells of a *35Spro-TTS-GFP* transgenic plant

**Supplementary Video S3** The vacuoles in unpollinated papilla cells of a *35Spro-TTS-GFP* transgenic plant with mock treatment

**Supplementary Video S4** The vacuoles in unpollinated papilla cells of a *35Spro-TTS-GFP* transgenic plant treated with 500 µM E-64d

**Supplementary Video S5** The vacuoles in unpollinated papilla cells of a *35Spro-TTS-GFP* transgenic plant treated with 500 µM PDMP

**Supplementary Video S6** The vacuoles in unpollinated papilla cells of a *35Spro-TTS-GFP* transgenic plant treated with 500 µM wortmannin

**Supplementary Video S7** The vacuoles in unpollinated papilla cells of a *35Spro-TTS-GFP* transgenic plant treated with 1 µM wortmannin

**Supplementary Video S8** The vacuoles in unpollinated papilla cells of a *35Spro-TTS-GFP* transgenic plant treated with 10 µM wortmannin

**Supplementary Video S9** The vacuoles in unpollinated papilla cells of a *35Spro-TTS-GFP* transgenic plant treated with 33 µM wortmannin

**Supplementary Video S10** The vacuoles in unpollinated papilla cells of a *35Spro-TTS-GFP* transgenic plant treated with 100 µM wortmannin

**Supplementary Video S11** The vacuoles in unpollinated papilla cells expressing *AtS1pro-TTS::GFP* in the *rop2-1* background

**Supplementary Video S12** The vacuoles in pollinated papilla cells expressing *AtS1pro-TTS::GFP* in the *rop2-1* background
